# Supplementary material for: Inflammatory factors and risk of lung adenocarcinoma: a Mendelian randomization study mediated by blood metabolites
Source: Front Endocrinol (Lausanne). 2024 Aug 27;15:1446863. doi: 10.3389/fendo.2024.1446863 (PMC11384989; doi:10.3389/fendo.2024.1446863)
Supplement: Supplementary file 1 [file DataSheet1.zip › supplementary files/S2/GCST90200274/sensitivity-analysis.pdf]

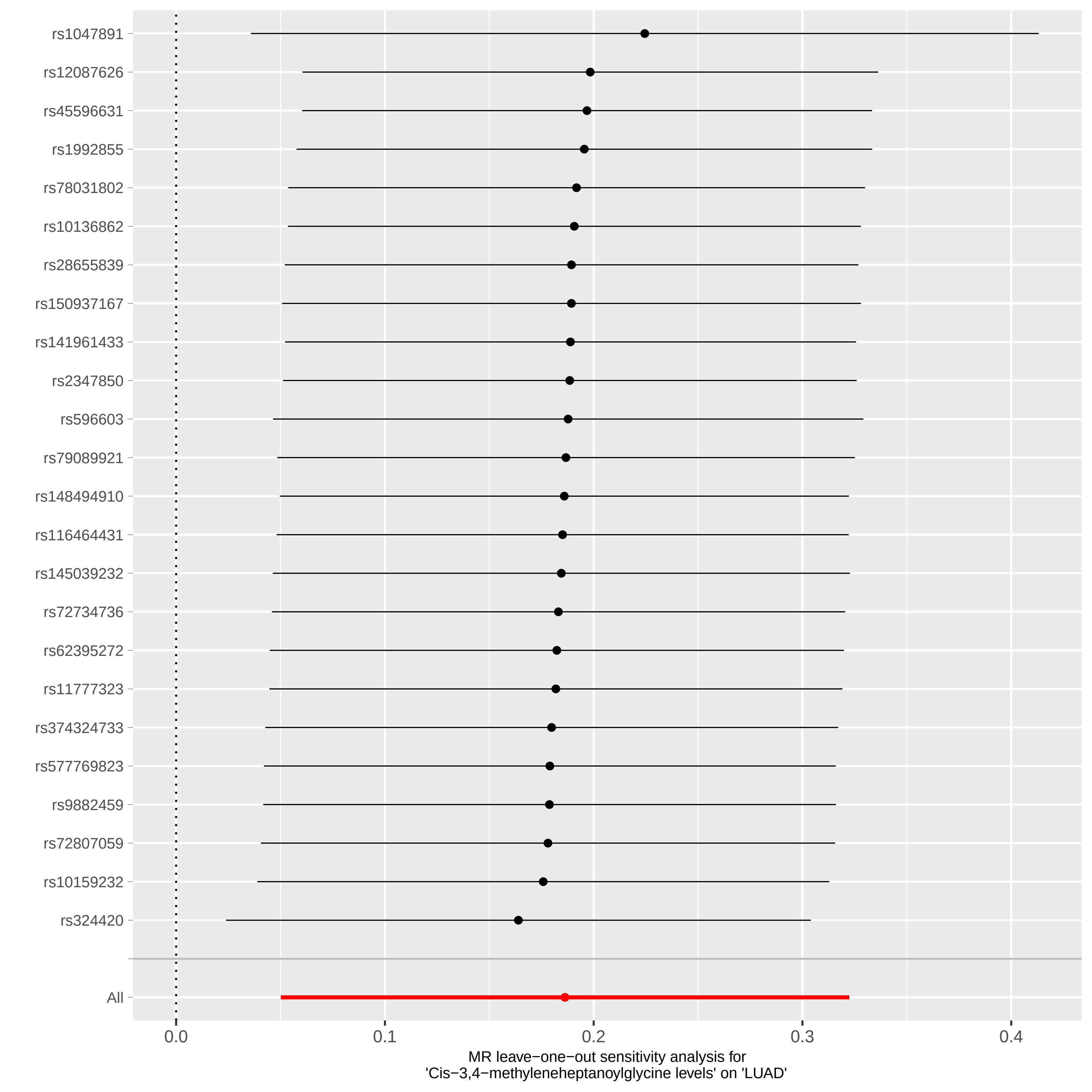
rs1047891
rs12087626
rs45596631
rs1992855
rs78031802
rs10136862
rs28655839
rs150937167
rs141961433
rs2347850
rs596603
rs79089921
rs148494910
rs116464431
rs145039232
rs72734736
rs62395272
rs11777323
rs374324733
rs577769823
rs9882459
rs72807059
rs10159232
rs324420
All
0.0
0.1
0.2
0.3
0.4
MR leave−one−out sensitivity analysis for
'Cis−3,4−methyleneheptanoylglycine levels' on 'LUAD'
